# Factors influencing students’ receptivity to formative feedback emerging from different assessment cultures

**DOI:** 10.1007/s40037-016-0297-x

**Published:** 2016-09-20

**Authors:** Christopher J. Harrison, Karen D. Könings, Elaine F. Dannefer, Lambert W. T. Schuwirth, Valerie Wass, Cees P. M. van der Vleuten

**Affiliations:** 1 Keele University School of Medicine, Keele, UK; 2 Faculty of Health, Medicine and Life Sciences, Maastricht University, Maastricht, The Netherlands; 3 Cleveland Clinic Lerner College of Medicine of Case Western Reserve University, Cleveland, USA; 4 Flinders Medical School, Adelaide, Australia

**Keywords:** Feedback, Summative assessment, Programmatic assessment

## Abstract

**Introduction:**

Feedback after assessment is essential to support the development of optimal performance, but often fails to reach its potential. Although different assessment cultures have been proposed, the impact of these cultures on students’ receptivity to feedback is unclear. This study aimed to explore factors which aid or hinder receptivity to feedback.

**Methods:**

Using a constructivist grounded theory approach, the authors conducted six focus groups in three medical schools, in three separate countries, with different institutional approaches to assessment, ranging from a traditional summative assessment structure to a fully implemented programmatic assessment system. The authors analyzed data iteratively, then identified and clarified key themes.

**Results:**

Helpful and counterproductive elements were identified within each school’s assessment system. Four principal themes emerged. Receptivity to feedback was enhanced by assessment cultures which promoted students’ agency, by the provision of authentic and relevant assessment, and by appropriate scaffolding to aid the interpretation of feedback. Provision of grades and comparative ranking provided a helpful external reference but appeared to hinder the promotion of excellence.

**Conclusions:**

This study has identified important factors emerging from different assessment cultures which, if addressed by programme designers, could enhance the learning potential of feedback following assessments. Students should be enabled to have greater control over assessment and feedback processes, which should be as authentic as possible. Effective long-term mentoring facilitates this process. The trend of curriculum change towards constructivism should now be mirrored in the assessment processes in order to enhance receptivity to feedback.

## What this paper adds

- This study seeks to address the problem with feedback after assessment, which often fails to reach its potential. The influence of assessment culture on receptivity to feedback has been unclear. This study demonstrates the benefits of moving away from a behaviouristic approach to assessment, based on punishment and rewards. It reveals the potential benefits of applying three constructivist principles to assessment: authenticity, empowering students with a more active role and gradual descaffolding to enable transformation towards a learning orientation.

## Introduction

For the development of optimal clinical performance, the importance of linking feedback with deliberate practice is generally acknowledged [1–3]. Regulatory authorities have called for feedback to feature strongly in medical training [4, 5]. Medical students demand more feedback after assessment [6, 7] and much advice is available for faculty on feedback delivery [8]. It is therefore surprising that learners sometimes neglect opportunities for feedback after assessment. In one study, 50 % of students failed to access feedback on an essay examination [9], and in another, students just achieving minimal competence in a summative Objective Structured Clinical Examination (OSCE) made least use of feedback [10].

Feedback may not reach its full potential in practice for several reasons. Faculty find it a complex process and fear being perceived as unkind to learners, as they struggle with conflicting aims of improving learners’ future performance and building their confidence. [11] Learners fear challenges to their own self-assessments, [12] often wanting feedback to boost their confidence, not correct deficiencies.[13] Feedback not aligned with a learner’s perception may be ignored [14, 15]. Medicine’s learning culture may also limit receptivity. In studies of music, athletics and teacher education, formative critical feedback was expected to push students towards excellence, whereas this was less expected within medicine [16–19].

To maximize learning from feedback, factors influencing its uptake in both high-stakes (summative) and low-stakes assessments should be explored. The summative assessment context has been shown to influence behaviour, emotions and cognitions fostering a reductionist approach which aims mainly to avoid failure and focuses attention on failing students who were ‘punished’ by resitting assessments [20]. In contrast, students who passed, even minimally, were ignored by faculty and felt little incentive to address their weaknesses.

Some medical schools have shifted towards ‘assessment for learning’ and programmatic assessment with a focus on multiple low-stakes assessments combined with rich, narrative-based feedback throughout the period of study [21–23]. However, implementing this approach in practice may not be straightforward. Recent studies have demonstrated that students may still regard the low-stakes assessments as summative hurdles to be overcome instead of learning opportunities [24, 25].It is therefore unclear how well these modifications to the assessment system promote learning from feedback.

Within these emerging differences in institutional assessment cultures, the impact on students’ reception to feedback appears complex. We developed a study to gain further insight. Our research question was: ‘what are the factors within medical schools’ assessment systems which aid or hinder student receptivity to feedback?’ We aimed to answer the question by exploring the experiences of students from three medical schools with different approaches to assessment and feedback.

## Method

### Context

We purposively selected three medical schools known to have different assessment and feedback systems. When considering which schools to include, we considered published reports as to whether the overall assessment programme was intended to promote ‘assessment for learning’ or ‘assessment of learning’. If the programme was predominantly one of ‘assessment of learning’, we nevertheless wanted an institution that explicitly provided feedback to students as this was a fundamental aspect of our research question. Cleveland Clinic Lerner College of Medicine, USA (School A) has a programmatic approach to assessment with narrative feedback. Keele University School of Medicine, UK (School B) has a mix of formative and summative assessments with both numerical and narrative feedback. The Physician-Clinical Investigator Programme at Maastricht University, Netherlands (School C) has a programmatic approach to assessment with both numerical and narrative feedback. More details of each school’s approach are listed in Table 1 and have been published elsewhere [10, 20, 25–29].

**Table 1:** Summary characteristics for schools used for research

	School A	School B	School C
	Cleveland Clinic Lerner College of Medicine, USA	Keele University School of Medicine, UK	Physician-Clinical Investigator Programme at Maastricht University, Netherlands
Programme Overview	5-year graduate entry	5-year undergraduate/graduate entry	4-year graduate-entry Masters
Students per year	32	130	50
Curriculum	Problem-based learning (PBL)	Mixed PBL with lectures	PBL
Assessment system	Programmatic approach to assessment	Mix of formative and summative assessment	Programmatic approach to assessment
Feedback system	Students receive formative narrative feedback from multiple sources. Grades and numerical scores are not used	Students receive feedback after all summative as well as formative assessments. Mix of numerical and narrative feedback	Mix of numerical and narrative feedback
Portfolio	Students compile a portfolio to interpret, analyze and triangulate the feedback received, with the aim of identifying personal strengths and weaknesses. They then write a reflective essay addressing their progress in meeting competencies, citing feedback as evidence	Students compile a portfolio comprising their personal reflections	Students collect all feedback and other evidence into a portfolio which is used for personal reflections
Mentoring	They meet regularly with a mentor, known as a physician advisor (PA). The PA is responsible for reviewing their formative portfolio	Each student is assigned a personal development tutor who meets them twice a year throughout the whole of the five year programme. This tutor is responsible for determining that the portfolio has been completed satisfactorily	Each student receives support from the same counsellor for all four years; the counsellor is not responsible for assessment decisions
Progression decision	Students are required to compile a summative portfolio, which is used to determine progression. This is assessed by a committee; the PA has no input into progression decisions	Progression determined solely by satisfactory performance in summative assessments	If a particular assessment demonstrates insufficient knowledge or skill acquisition, the student is required to perform further assessments in order to demonstrate satisfactory competence. The assessment information and feedback in the portfolio is evaluated at the end of the year by an independent portfolio assessment committee and used for the high-stakes promotion decision

### Data collection

We chose focus groups in order to seek a range of views and to enable expressed ideas to be developed through interaction between participants. We conducted six focus groups in April to June 2014 (two at each school) to gather the perceptions of the student cohort on their feedback at different points in their studies. A faculty member from the local school, unconnected with the research project, recruited students by email. A convenience sampling approach was taken based on the students’ availability at pre-determined times. Incentives to participate were not offered. To encourage discussion of potentially challenging areas, each group deliberately consisted of a small number of students from a single year. More details of the groups are shown in Table 2. The order in which the groups took place was determined by timetabling constraints: B1, A1, A2, C1, C2, B2. Logistical issues prevented the organization of further groups in order to attempt to reach data saturation on each site. All students from Schools A and B spoke English as a first language; students at School C spoke English as a second language, but all were fluent and had no language difficulties.

**Table 2:** Participants in each focus group

	School A		School B		School C	
Focus group	1	2	1	2	1	2
Year group and part of the course	1 Basic science	4 Clinical	3 Early Clinical	4 Clinical	1 Basic science	4 Clinical
Number of students	4	3	5	5	5	5

The focus groups took place in the respective institutions with a single facilitator (CH) and lasted 60–120 min. A semi-structured approach was underpinned by open-ended questions designed to elicit students’ perceptions of their institution’s assessment system, their interpretation and use of feedback and any effect of grades on their aspiration to excellence. Questioning evolved according to the participants’ responses. We recorded and transcribed discussions maintaining student anonymity.

### Data analysis

We used a constructivist grounded theory approach. This approach to qualitative data analysis encompasses the notion that interpretation of the data is co-constructed by both researchers and participants [30]. By studying the experiences and perspectives of the participants described in the transcripts, we aimed to identify thematic categories of factors that aid or hinder the uptake of feedback. Consistent with grounded theory, analysis occurred alongside data collection and was able to inform the questioning in the later groups. The lead author (CH) coded all transcripts in order to organize the data and identify key themes and concepts. A second researcher (VW) separately coded three transcripts (one from each school). There was close agreement; minor discrepancies were discussed and could be quickly resolved. At regular intervals, the research team conducted Skype teleconferences to refine the conceptual analysis. As our analysis framework assumes that data are co-constructed by interactions between researchers and participants, we provide the following contextual information: CH, LS, VW are medical doctors with a major involvement in medical education research and development; CvdV, KK have backgrounds in psychology and ED in sociology with a major involvement in medical education research and development.

## Results

Four principal themes emerged: (1) Personal agency; (2) Authenticity and relevance of assessment; (3) Grades and comparative ranking; (4) Scaffolding of feedback. They are described below, with illustrative participant quotes to illustrate the themes.

### Personal agency

Factors within the assessment systems across all schools promoted, or hindered, students’ personal agency, their capacity to act and make choices using their personal abilities within the constraints and possibilities of their context. Agency was promoted by providing choice, either within compulsory assessments or by providing optional assessments, which enabled students to demonstrate knowledge acquisition to themselves and their tutors. In contrast, compulsory multiple-choice question papers reduced personal agency by preventing students from fully demonstrating their knowledge; the questions were perceived to focus on specific ‘random’ facts.

*I’m often frustrated with the questions because I think, ‘Well, I’m actually good in this field but this one particular question, I don’t know.’ (School C, Focus Group (FG)1)*

Students discounted much of the feedback as irrelevant for future learning or assessments. Feedback methods enabling students to act independently were more conducive to supporting personal agency than those restricting autonomy. For example, online questions and feedback, combined with the ability to revisit these at any time, were preferred to didactic tutor-led feedback sessions delivered in a group setting without provision to revisit later. Providing significant autonomy within the assessment and feedback systems fostered an aspiration towards excellence:

*It [the School] really lets you….truly reach and seek that excellence because it gives you that time that you can invest in whatever you deem to be important. (School A, FG1)*

By contrast, an assessment system dominated by high-stakes assessments discouraged aspiration to improve:

*I think sometimes exams can be limiting because you say, ‘Oh, I don’t need to know that for an exam.’ ……that stifles learning sometimes. (School B, FG1)*

Students felt in control if feedback from low-stakes assessments appeared predictive of future performance in high-stakes assessments. The feeling was the opposite if low-stakes assessments were seen as too dissimilar from the ‘real thing’.

Institutional requirements for assessments (e. g. formatting rules for completing a portfolio) limited agency and provoked much frustration and risked devaluing learning. Students sometimes tried to subvert the marking criteria to maximize learning, demonstrating tension between student agency and institutional control.

*Instead of looking at what they wanted I just went on a tangent and wrote. And then that for me I was like, I know I might not get a good mark. I might not even pass, but this is actually reflective. That’s going to be useful for me in the future to look back at. (School B, FG1)*

Similarly, agency was threatened when institutions set criteria for selecting specific items from overall aggregated feedback to demonstrate progress. Students perceived being forced to include critical feedback, at the expense of detailed, personal and positive feedback, even if it was unhelpful, bland and generic. They felt forced to play the ‘assessment game’.

*I had feedback, ‘You’re not saying enough.’ But on the other hand I got feedback, ‘But the quality of what you’re saying is very high.’ But I was always forced to make some goals from, ‘You’re not saying enough,’ so I was forced to say more. (School C, FG1)*

Students recognized that autonomy should have limits. The safety of future patients necessitated that assessment processes identify students’ weaker areas or flaws in self-assessment. Standardization of assessments was generally accepted, even though it limited autonomy, but acceptance was much lower if it was perceived to lack immediate relevance to their future role as doctors.

*If we’re going to do a critical appraisal of qualitative research, why not get people to find their own piece? So if you were doing paediatrics at the time, you could have found a paediatric paper that was relevant rather than writing about bottled water because it’s not got any clinical relevance to what we’re doing. (School B, FG1)*

## Authenticity and relevance of assessment

Many assessments were seen to lack authenticity or direct relevance to students’ future work as doctors. Feedback was often ignored if assessments were perceived as irrelevant. There was a belief that it was necessary to say and do certain things to pass a clinical skills assessment (such as an OSCE); these were different from what would be done in real-life clinical practice. Feedback given on wards before an OSCE was often ignored and perceived to harm the chances of passing. In contrast, an assessment system which relied only on feedback within the clinical workplace, in the absence of high-stakes OSCEs, avoided giving students mixed messages. It allowed them to concentrate on feedback they received. Recognition that learning, and developing clinical competence, was an evolving process continuing after graduation appeared particularly helpful:

*It’s kind of comforting that this process [of ward-based feedback] continues as we’re residents and that we learn at a similar rate that we’re learning here in medical school. (School A, FG2)*

### Grades and comparative ranking

Grades and comparative student rankings within the cohort had mixed effects on receptivity to feedback. Grades reassured students they were achieving the standards required for qualification:

*I think it’s like comparing my grades to what they think is necessary. So the necessary level of knowledge to be a doctor, is my knowledge lower than that? Is it higher than that? Is it about right? (School B, FG1)*

Grades provided some clarity on the expected level of performance and avoided the potential for narrative feedback to mislead:

*Well, I mean, you can get a warm and fuzzy description and be four out of ten. If you get a four then you know you need to do something to get to the six that is required to make the grade. I would much rather know that I need to work harder than have a warm and fuzzy feeling. (School B, FG2)*

Information comparing performance with peers was an additional stimulus to take feedback seriously:

*If I got a ranking [position relative to peers] of 50 on my MCQs and a ranking of 20 on my KFPs [Key Feature Problems], then I know I need to go and work on my MCQs. (School B, FG1*)

Grades following summative, end-of-year assessments gave sufficient information; more detailed feedback was seen as superfluous:

*So what was on our Year 2 paper – most of it is irrelevant to what’s going to be on our third-year paper. So to give us feedback is pointless. We’ve got a grade. That’s all we need to know. (School B, FG1)*

At other times within a programme, grades without narrative feedback failed to provide sufficient information or motivation to stimulate improvement. At one school, so many students were graded ‘at the level expected’ that it was felt futile to aspire towards excellence.

*And for these reports, ….. I don’t really feel stimulated to do my very best to get a good grade because I know it will just be on the threshold and I’ll be fine. (School C, FG1)*

The transition from a grade-based system in a previous programme to one based on narrative feedback without grades presented a challenge for students to then find ways of checking they were ‘on track’.

*You don’t have that oh, I got an A. Okay, you know, I can feel good about myself. It’s just…you don’t have that external validation. (School A, FG2*)

As students adjusted to this new assessment model, they became less dependent on validating labels and focussed more on preparation for practice:

*You can see that this is preparing you for real life when you don’t get an A every time you go. (School A, FG2)*

As the transition continued, the absence of grading or ranking forced students to take a more nuanced view of their peers’ strengths and weaknesses. This gave them more freedom in using the feedback while preserving their own self-esteem. The lack of reassurance from ‘good enough’ grades incentivized students to aim for excellence:

*We don’t know what the bare minimum is. You better have a slam-dunk to make sure. (School A, FG1)*

The tendency for tutors at all schools to provide vague positive feedback frustrated students; it failed to meet aspirations to improve performance.

*It means very little to me to always get these ‘great job, great job, great job’ versus someone who is trying to find ways to help me get better. (School A, FG2)*

It was also commonly perceived that tutors feared giving critical feedback. Students at School A described how they had actively challenged tutors for more constructively critical feedback. There was little evidence of feedback-seeking behaviour at the other sites:

*They have to be prompted because they’re afraid to write something bad in our feedback, but it’s necessary. (School A, FG2)*

## Scaffolding of feedback

Scaffolding, as provided by mentors, played a significant role in assisting, or occasionally hindering, receptivity to feedback. Successful mentoring helped students interpret feedback while allowing them to remain in control. Agency was promoted by reducing the scaffolding as the course progressed to prepare students for a working environment reducing dependency on feedback:

*My PA [mentor]… [said]… the whole point of this system is that at some point in your career, you’re not going to have people giving you monthly evaluations necessarily, ……you’re supposed to be able to self-identify this and reflect and then do that. (School A, FG2)*

A student mentoring system which provided long-term supervision (over several years), and allowed mentors to see the student’s entire feedback, enabled trust to develop. Awareness of previous feedback aided interpretation of new information:

*They also know all your previous feedback, so they can kind of help you contextualize it. (School A, FG2)*

Organizing the support systems in this way enabled mentors to safely challenge students’ inaccurate self-assessments:

*I tend to be very hard on myself to begin with, and so I would actually meet with my PA, and she goes, … this is what I’m seeing in the evidence, and this is what you’re saying and they don’t really match. (School A, FG2)*

Scaffolding did not always enhance students’ agency. Mentors could become paternalistic and decide what feedback to exclude from the portfolio:

*They’ve got all different opinions about what’s good. So then one counsellor comes and says, ‘No, you should change this.’ And then next you get another counsellor who’s also going to check your portfolio and then suddenly it’s all wrong and you’ve got to change it back. (School C, FG2)*

## Discussion

We aimed to explore factors within different medical school assessment systems affecting student receptivity to feedback and have demonstrated several important factors. Offering students choice and independence within assessment systems promoted receptivity to feedback. Assessments perceived to lack relevance and authenticity hindered openness to feedback. By contrast, assessments and feedback were valued if authentically aligned to their future work. Grades and comparative ranking marks superficially reassured students they were ‘good enough’. Not providing grades caused initial uncertainty, but later promoted more authentic recognition that both self and peers had a complex set of strengths and weaknesses which could not easily be labelled. This encouraged an aspiration to excellence. Long-term mentor relationships assisted both students’ interpretation of feedback and enabled inaccurate self-assessment to be challenged, as long as mentors avoided paternalistic attitudes which reduced students’ agency. Students ignored feedback lacking credibility or quality.

Previous studies have shown the influence of learning culture in modulating receptivity to feedback. In music and sport, a long-term close working relationship with a mentor enables critical feedback to be accepted within an atmosphere of trust [19]. In longitudinal integrated clerkships, authentic assessment and feedback and a supportive mentoring relationship can promote learning [31]. To our knowledge, the importance of enabling greater student choice and independence within assessment processes to foster greater receptivity to feedback has not previously been described.

### Implications for medical education

In recent decades behaviourist approaches to learning, relying on passive knowledge acquisition, have moved towards constructivist approaches and active learning strategies [32]. By contrast, the fundamental approach towards assessment has remained behaviourist. Students are rewarded for passing or punished for failing. Many institutions, including those with active learning approaches, have maintained assessments that reward superficial learning strategies, which students unsurprisingly adopt [33, 34]. Although more constructivist principles for assessment have been requested [35–37], implementation has been limited. Our study demonstrates the potential benefits of three key constructivist principles: (i) improving the authenticity of the assessment, (ii) empowering students with a more active role and (iii) gradual descaffolding to enable transformation from a performance orientation towards a learning orientation. Active involvement in assessment and feedback processes is, however, neither easily achieved nor a panacea, and the role of the authenticity of assessments and the credibility of the mentor/coach as a knowledgeable other are important factors in this transformation. There are clearly patient safety risks if students are given excessive choice within assessments too early in the curriculum and the ensuing liberty to ignore uncomfortable feedback. Nevertheless, we argue that promoting and gradually developing a culture of receptivity to feedback will ultimately benefit patient safety.

A long-term mentoring relationship appears essential for feedback provision to stimulate effective learning. It enables trust to develop in a safe environment in which learners can be challenged. Although such relationships are common in other professions, medical education often involves multiple short-term clerkships [19, 31]. As with the transition to problem-based learning, mentors may struggle to adopt facilitative rather than paternalistic approaches [38].

The study was not designed to directly compare the programmatic approach to assessment with the more traditional summative assessment approach. Within each school’s assessment system we identified both helpful and counterproductive elements which impacted on receptivity to feedback. The generalization of our findings therefore does not come from stable factors that are supposed to be true in all contexts; rather our findings serve to better understand the driving forces to enable adaptability of this knowledge in the design of assessment in various contexts. Designers of assessment and feedback programmes could benefit from incorporating the more helpful elements in their programmes.

### Limitations

Our study has some limitations. Only the participants’ perspectives could be interpreted by the authors to co-construct meaning which may mean that certain perspectives may be over-represented and others may be under-represented. We do not claim to have achieved data saturation over the six focus groups. So although we think that the themes emerging from our study are important and make good sense, we are confident that there are other interesting themes that would emerge from future replications of our study. As such the combination of such studies would serve to complete the whole picture. A further support for the sensibility of our co-constructed conclusions is that they resonate with the existing feedback literature and yet allow a more focused ‘lens’ on the interactional factors between the learner and their learning context. The decision to sample different year groups was made on good grounds, but the differences between curricula may also have led to under-representation of certain perceptions, so again we want to stress that we do not assume to have been able to provide a complete picture of all themes. To triangulate students’ self-reported perceptions, it would be important in future studies to explore the perceptions of mentors and other faculty regarding the students’ receptivity to feedback.

### Suggestions for further research

Our results suggest several opportunities for further investigation. While it may be clear that there is a need to gradually shift the control over the assessment process from teacher to learner it is important to better understand the factors that could slow down or accelerate this process. Future studies seeking to understand these factors would be highly informative for assessment design. As stated above it would also be important to replicate our study in a different context to establish whether important themes regarding the uptake of feedback would be detected that our study did not find. Finally, research into the conceptualizations of faculty is of paramount importance to allow for triangulation of these themes.

## Conclusions

This study has sought to understand the key elements within an assessment system which influence receptivity to feedback. Whether a medical school employs a summative assessment-based structure or adopts the principles of programmatic assessment, we should strive to make the assessments as authentic as possible, consider carefully the benefits and risks of awarding grades and use long-term mentoring to enable students to be both supported and challenged by the feedback they receive. Students perceive that they benefit from a greater degree of control over assessment and feedback processes. The trend of curriculum change towards constructivism should now be mirrored in the assessment processes. Though challenging we believe this can be achieved.
